# Supplementary material for: Oral Health-Related Quality of Life in Different Subtypes of Ehlers-Danlos Syndrome
Source: Int J Environ Res Public Health. 2023 Jan 26;20(3):2218. doi: 10.3390/ijerph20032218 (PMC9916345; doi:10.3390/ijerph20032218)
Supplement: Supplementary file 1 [file ijerph-20-02218-s001.zip › ijerph-2122009-supplementary/Supplementary Table S1.pdf]

Supplementary Table S1

Distribution of responses (%) of summary OHIP-14 items for all Ehlers-Danlos syndromes subtypes; translated items of the German short form of the Oral Health Impact Profile (OHIP-14G).

OHIP-14	Never (%)	Hardly Ever (%)	Occasionally (%)	Often (%)	Very Often (%)
**Have you had any problems with your teeth, mouth, or dentures in the past month…**					
…making it difficult to pronounce certain words?	151 (51.1)	58 (19.7)	54 (18.3)	26 (8.8)	6 (2.0)
…that made you feel that your sense of taste was impaired?	162 (54.9)	54 (18.3)	40 (13.6)	25 (8.5)	14 (4.7)
…that gave you the impression that your life, in general, was less satisfying?	111 (37.6)	63 (21.4)	74 (25.1)	33 (11.2)	14 (4.7)
…making it difficult to relax?	61 (20.7)	38 (12.9)	96 (32.5)	64 (21.7)	36 (12.2)
**Has it often happened in the past month due to problems with your teeth, mouth, or dentures…**					
…that you felt tense?	54 (18.3)	48 (16.3)	95 (32.2)	64 (21.7)	34 (11.5)
…that you had to interrupt meals?	99 (33.6)	71 (24.1)	68 (23.1)	34 (11.5)	23 (7.8)
…that you were uncomfortable eating certain foods?	69 (23.4)	42 (14.2)	75 (25.4)	61 (20.7)	48 (16.3)
…that you have been a bit irritable with other people?	111 (37.6)	66 (22.4)	81 (27.5)	29 (9.8)	8 (2.7)
…that you had difficulty doing your usual jobs?	102 (34.6)	77 (26.1)	79 (26.8)	25 (8.5)	12 (4.1)
…that you were completely unable to do anything?	171 (58.0)	69 (23.4)	39 (13.2)	10 (3.4)	6 (2.0)
…that you have been a bit embarrassed?	158 (53.6)	48 (16.3)	44 (14.9)	28 (9.5)	17 (5.8)
…that your diet has been unsatisfactory?	127 (43.1)	52 (17.6)	66 (22.4)	29 (9.8)	21 (7.1)
**In the past month, did you have…**					
…any pain in your mouth?	23 (7.8)	45 (15.3)	95 (32.2)	72 (24.4)	60 (20.3)
…a feeling of uncertainty about your teeth, mouth, or dentures?	72 (24.4)	67 (22.7)	52 (17.6)	56 (19.0)	48 (16.3)
