# Supplementary material for: Oral Health-Related Quality of Life in Different Subtypes of Ehlers-Danlos Syndrome
Source: Int J Environ Res Public Health. 2023 Jan 26;20(3):2218. doi: 10.3390/ijerph20032218 (PMC9916345; doi:10.3390/ijerph20032218)
Supplement: Supplementary file 1 [file ijerph-20-02218-s001.zip › ijerph-2122009-supplementary/Supplementary Table S2.pdf]

Supplementary Table S2

Sensitivity analysis between subtypes. P-values for each column were calculated using a Kruskal-Wallis Test, leaving out one subtype at a time, to assess its impact.

	n	Median	IQR	Mean	SD	*p*-value
without cEDS	266	19	18	19.7	12.2	0.091
without clEDS	288	19	19	19.4	12.3	0.163
without cvEDS	294	19	18	19.6	12.3	0.079
without vEDS	277	19	19	19.8	12.3	0.149
without hEDS	65	20	23	19.9	13.5	0.91
without aEDS	293	19	18	19.6	12.3	0.079
without kEDS	293	19	19	19.5	12.3	0.146
without BCS	294	19	18	19.5	12.2	0.181
without mEDS	293	19	19	19.4	12.2	0.259
without pEDS	292	19	18	19.5	12.3	0.082
