# Supplementary material for: Oral Health-Related Quality of Life in Different Subtypes of Ehlers-Danlos Syndrome
Source: Int J Environ Res Public Health. 2023 Jan 26;20(3):2218. doi: 10.3390/ijerph20032218 (PMC9916345; doi:10.3390/ijerph20032218)
Supplement: Supplementary file 1 [file ijerph-20-02218-s001.zip › ijerph-2122009-supplementary/Supplementary Table S3.pdf]

Supplementary Table S3

Distribution of responses (%) of individual OHIP-14 items for all Ehlers-Danlos syndromes subtypes

Classic EDS

OHIP-14	Never	Hardly ever	Occasionally	Often	Very often
Have you had any problems with your teeth, mouth or dentures in the past month…					
…making it difficult to pronounce certain words?	16 (55.2)	4 (13.8)	6 (20.7)	2 (6.9)	1 (3.4)
…that made you feel that your sense of taste was impaired?	20 (69.0)	2 (6.9)	2 (6.9)	4 (13.8)	1 (3.4)
…that gave you the impression that your life in general was less satisfying?	12 (41.4)	7 (24.1)	6 (20.7)	2 (6.9)	2 (6.9)
…making it difficult to relax?	6 (20.7)	4 (13.8)	9 (31.0)	9 (31.0)	1 (3.4)
Has it often happened in the past month due to problems with your teeth, mouth or dentures…					
…that you felt tense?	4 (13.8)	3 (10.3)	11 (37.9)	10 (34.5)	1 (3.4)
…that you had to interrupt meals?	13 (44.8)	4 (13.8)	7 (24.1)	3 (10.3)	2 (6.9)
…that you were uncomfortable eating certain foods?	9 (31.0)	1 (3.4)	9 (31.0)	3 (10.3)	7 (24.1)
…that you have been a bit irritable with other people?	9 (31.0)	10 (34.5)	6 (20.7)	3 (10.3)	1 (3.4)
…that you had difficulty doing your usual jobs?	13 (44.8)	6 (20.7)	6 (20.7)	3 (10.3)	1 (3.4)
…that you were completely unable to do anything?	18 (62.1)	4 (13.8)	4 (13.8)	3 (10.3)	0 (0.0)
…that you have been a bit embarrassed?	18 (62.1)	5 (17.2)	4 (13.8)	2 (6.9)	0 (0.0)
…that your diet has been unsatisfactory?	12 (41.4)	6 (20.7)	6 (20.7)	3 (10.3)	2 (6.9)
In the past month, did you have…					
…any pain in your mouth?	7 (24.1)	5 (17.2)	5 (17.2)	8 (27.6)	4 (13.8)
…a feeling of uncertainty about your teeth, mouth, or dentures?	8 (27.6)	8 (27.6)	7 (24.1)	3 (10.3)	3 (10.3)

Classic-like EDS

OHIP-14	Never	Hardly ever	Occasionally	Often	Very often
Have you had any problems with your teeth, mouth or dentures in the past month…					
…making it difficult to pronounce certain words?	4 (57.1	2 (28.6)	0 0.0)	0 (0.0)	1 (14.3)
…that made you feel that your sense of taste was impaired?	3 (42.9)	1 (14.3)	2 (28.6)	0 (0.0)	1 (14.3)
…that gave you the impression that your life in general was less satisfying?	3 (42.9)	0 (0.0)	0 (0.0)	3 (42.9)	1 (14.3)
…making it difficult to relax?	0 (0.0)	1 (13.3)	4 (57.1)	2 (28.6)	0 (0.0)
Has it often happened in the past month due to problems with your teeth, mouth or dentures…					
…that you felt tense?	1 (14.3)	0 (0.0)	2 (28.6)	2 (28.6)	2 (28.6)
…that you had to interrupt meals?	1 (14.3)	2 (28.6)	2 (28.6)	1 (14.3)	1 (14.3)
…that you were uncomfortable eating certain foods?	1 (14.3)	0 (0.0)	1 (14.3)	1 (14.3)	4 (57.1)
…that you have been a bit irritable with other people?	3 (42.9)	1 (14.3)	1 (14.3)	2 (28.6)	0 (0.0)
…that you had difficulty doing your usual jobs?	2 (28.6)	1 (14.3)	1 (14.3)	1 (14.3)	2 (28.6)
…that you were completely unable to do anything?	3 (42.9)	2 (28.6)	1 (14.3)	1 (14.3)	0 (0.0)
…that you have been a bit embarrassed?	2 (28.6)	1 (14.3)	2 (28.6)	1 (14.3)	1 (14.3)
…that your diet has been unsatisfactory?	2 (28.6)	1 (14.3)	3 (42.9)	1 (14.3)	0 (0.0)
In the past month, did you have…					
…any pain in your mouth?	0 (0.0)	0 (0.0)	2 (28.6)	2 (28.6)	3 (42.9)
…a feeling of uncertainty about your teeth, mouth, or dentures?	1 (14.3)	2 (28.6)	0 (0.0)	2 (28.6)	2 (28.6)

Cardiac-valvular EDS

OHIP-14	Never	Hardly ever	Occasionally	Often	Very often
Have you had any problems with your teeth, mouth or dentures in the past month…					
…making it difficult to pronounce certain words?	1 (100.0)	0 (0.0)	0 (0.0)	0 (0.0)	0 (0.0)
…that made you feel that your sense of taste was impaired?	1 (100.0)	0 (0.0)	0 (0.0)	0 (0.0)	0 (0.0)
…that gave you the impression that your life in general was less satisfying?	0 (0.0)	0 (0.0)	1 (100.0)	0 (0.0)	0 (0.0)
…making it difficult to relax?	0 (0.0)	0 (0.0)	1 (100.0)	0 (0.0)	0 (0.0)
Has it often happened in the past month due to problems with your teeth, mouth or dentures…					
…that you felt tense?	0 (0.0)	0 (0.0)	0 (0.0)	1 (100.0)	0 (0.0)
…that you had to interrupt meals?	0 (0.0)	1 (100.0)	0 (0.0)	0 (0.0)	0 (0.0)
…that you were uncomfortable eating certain foods?	0 (0.0)	1 (100.0)	0 (0.0)	0 (0.0)	0 (0.0)
…that you have been a bit irritable with other people?	0 (0.0)	0 (0.0)	1 (100.0)	0 (0.0)	0 (0.0)
…that you had difficulty doing your usual jobs?	0 (0.0)	0 (0.0)	0 (0.0)	1 (100.0)	0 (0.0)
…that you were completely unable to do anything?	0 (0.0)	1 (100.0)	0 (0.0)	0 (0.0)	0 (0.0)
…that you have been a bit embarrassed?	0 (0.0)	0 (0.0)	1 (100.0)	0 (0.0)	0 (0.0)
…that your diet has been unsatisfactory?	1 (100.0)	0 (0.0)	0 (0.0)	0 (0.0)	0 (0.0)
In the past month, did you have…					
…any pain in your mouth?	0 (0.0)	1 (100.0)	0 (0.0)	0 (0.0)	0 (0.0)
…a feeling of uncertainty about your teeth, mouth, or dentures?	0 (0.0)	0 (0.0)	0 (0.0)	1 (100.0)	0 (0.0)

Vascular EDS

OHIP-14	Never	Hardly ever	Occasionally	Often	Very often
Have you had any problems with your teeth, mouth or dentures in the past month…					
…making it difficult to pronounce certain words?	10 (55.6)	4 (22.2)	1 (5.6)	3 (16.7)	0 (0.0)
…that made you feel that your sense of taste was impaired?	9 (50.0)	2 (11.1)	4 (22.2)	3 (16.7)	0 (0.0)
…that gave you the impression that your life in general was less satisfying?	5 (27.8)	3 (16.7)	9 (50.0)	0 (0.0)	1 (5.6)
…making it difficult to relax?	6 (33.3)	3 (16.7)	5 (27.8)	3 (16.7)	1 (5.6)
Has it often happened in the past month due to problems with your teeth, mouth or dentures…					
…that you felt tense?	8 (44.4)	2 (11.1)	5 (27.8)	3 (16.7)	0 (0.0)
…that you had to interrupt meals?	9 (50.0)	5 (27.8)	1 (5.6)	2 (11.1)	1 (5.6)
…that you were uncomfortable eating certain foods?	9 (50.0)	1 (5.6)	4 (22.2)	3 (16.7)	1 (5.6)
…that you have been a bit irritable with other people?	10 (55.6)	4 (22.2)	4 (22.2)	0 (0.0)	0 (0.0)
…that you had difficulty doing your usual jobs?	9 (50.0)	5 (27.8)	3 (16.7)	0 (0.0)	1 (5.6)
…that you were completely unable to do anything?	12 (66.7)	3 (16.7)	2 (11.1)	0 (0.0)	1 (5.6)
…that you have been a bit embarrassed?	11 (61.1)	4 (22.2)	1 (5.6)	1 (5.6)	1 (5.6)
…that your diet has been unsatisfactory?	11 (61.1)	3 (16.7)	3 (16.7)	1 (5.6)	0 (0.0)
In the past month, did you have…					
…any pain in your mouth?	4 (22.2)	1 (5.6)	7 (38.9)	4 (22.2)	2 (11.1)
…a feeling of uncertainty about your teeth, mouth, or dentures?	5 (27.8)	3 (16.7)	5 (27.8)	3 (16.7)	2 (11.1)

Hypermobile EDS

OHIP-14	Never	Hardly ever	Occasionally	Often	Very often
Have you had any problems with your teeth, mouth or dentures in the past month…					
…making it difficult to pronounce certain words?	115 (50.0)	46 (20.0)	47 (20.4)	18 (7.8)	4 (1.7)
…that made you feel that your sense of taste was impaired?	124 (53.9)	48 (20.9)	30 (13.0)	16 (7.0)	12 (5.2)
…that gave you the impression that your life in general was less satisfying?	89 (38.7)	53 (23.0)	55 (23.9)	24 (10.4)	9 (3.9)
…making it difficult to relax?	48 (20.9)	29 (12.6)	77 (33.5)	45 (19.6)	31 (13.5)
Has it often happened in the past month due to problems with your teeth, mouth or dentures…					
…that you felt tense?	39 (17.0)	42 (18.3)	75 (32.6)	46 (20.0)	28 (12.2)
…that you had to interrupt meals?	74 (32.2)	56 (24.3)	58 (25.2)	25 (10.9)	17 (7.4)
…that you were uncomfortable eating certain foods?	48 (20.9)	37 (16.1)	60 (26.1)	52 (22.6)	33 (14.3)
…that you have been a bit irritable with other people?	87 (37.8)	50 (21.7)	66 (28.7)	21 (9.1)	6 (2.6)
…that you had difficulty doing your usual jobs?	76 (33.0)	64 (27.8)	67 (29.1)	16 (7.0)	7 (3.0)
…that you were completely unable to do anything?	134 (58.3)	57 (24.8)	31 (13.5)	3 (1.3)	5 (2.2)
…that you have been a bit embarrassed?	124 (53.9)	36 (15.7)	35 (15.2)	22 (9.6)	13 (5.7)
…that your diet has been unsatisfactory?	98 (42.6)	40 (17.4)	52 (22.6)	24 (10.4)	16 (7.0)
In the past month, did you have…					
…any pain in your mouth?	12 (5.2)	37 (16.1)	80 (34.8)	56 (24.3)	45 (19.6)
…a feeling of uncertainty about your teeth, mouth, or dentures?	57 (24.8)	54 (23.5)	38 (16.5)	45 (19.6)	36 (15.7)

Arthrochalasia EDS

OHIP-14	Never	Hardly ever	Occasionally	Often	Very often
Have you had any problems with your teeth, mouth or dentures in the past month…					
…making it difficult to pronounce certain words?	0 (0.0)	1 (50.0)	0 (0.0)	1 (50.0)	0 (0.0)
…that made you feel that your sense of taste was impaired?	1 (50.0)	0 (0.0)	1 (50.0)	0 (0.0)	0 (0.0)
…that gave you the impression that your life in general was less satisfying?	1 (50.0)	0 (0.0)	0 (0.0)	1 (50.0)	0 (0.0)
…making it difficult to relax?	0 (0.0)	1 (50.0)	0 (0.0)	1 (50.0)	0 (0.0)
Has it often happened in the past month due to problems with your teeth, mouth or dentures…					
…that you felt tense?	1 (50.0)	0 (0.0)	1 (50.0)	0 (0.0)	0 (0.0)
…that you had to interrupt meals?	1 (50.0)	1 (50.0)	0 (0.0)	0 (0.0)	0 (0.0)
…that you were uncomfortable eating certain foods?	1 (50.0)	1 (50.0)	0 (0.0)	0 (0.0)	0 (0.0)
…that you have been a bit irritable with other people?	1 (50.0)	0 (0.0)	1 (50.0)	0 (0.0)	0 (0.0)
…that you had difficulty doing your usual jobs?	1 (50.0)	0 (0.0)	0 (0.0)	1 (50.0)	0 (0.0)
…that you were completely unable to do anything?	1 (50.0)	0 (0.0)	1 (50.0)	0 (0.0)	0 (0.0)
…that you have been a bit embarrassed?	1 (50.0)	0 (0.0)	1 (50.0)	0 (0.0)	0 (0.0)
…that your diet has been unsatisfactory?	1 (50.0)	1 (50.0)	0 (0.0)	0 (0.0)	0 (0.0)
In the past month, did you have…					
…any pain in your mouth?	0 (0.0)	1 (50.0)	0 (0.0)	1 (50.0)	0 (0.0)
…a feeling of uncertainty about your teeth, mouth, or dentures?	1 (50.0)	0 (0.0)	0 (0.0)	1 (50.0)	0 (0.0)

Kyphoscoliotic EDS

OHIP-14	Never	Hardly ever	Occasio nally	Often	Very often
Have you had any problems with your teeth, mouth or dentures in the past month…					
…making it difficult to pronounce certain words?	0 (0.0)	1 (50.0)	0 (0.0)	1 (50.0)	0 (0.0)
…that made you feel that your sense of taste was impaired?	1 (50.0)	1 (50.0)	0 (0.0)	0 (0.0)	0 (0.0)
…that gave you the impression that your life in general was less satisfying?	0 (0.0)	0 (0.0)	2 (100.0)	0 (0.0)	0 (0.0)
…making it difficult to relax?	0 (0.0)	0 (0.0)	0 (0.0)	1 (50.0)	1 (50.0)
Has it often happened in the past month due to problems with your teeth, mouth or dentures…					
…that you felt tense?	0 (0.0)	1 (50.0)	1 (50.0)	0 (0.0)	0 (0.0)
…that you had to interrupt meals?	0 (0.0)	1 (50.0)	0 (0.0)	1 (50.0)	0 (0.0)
…that you were uncomfortable eating certain foods?	0 (0.0)	1 (50.0)	0 (0.0)	0 (0.0)	1 (50.0)
…that you have been a bit irritable with other people?	0 (0.0)	1 (50.0)	1 (50.0)	0 (0.0)	0 (0.0)
…that you had difficulty doing your usual jobs?	0 (0.0)	1 (50.0)	1 (50.0)	0 (0.0)	0 (0.0)
…that you were completely unable to do anything?	0 (0.0)	1 (100.0)	0 (0.0)	0 (0.0)	0 (0.0)
…that you have been a bit embarrassed?	0 (0.0)	0 (0.0)	0 (0.0)	1 (50.0)	1 (50.0)
…that your diet has been unsatisfactory?	0 (0.0)	1 (50.0)	0 (0.0)	0 (0.0)	1 (50.0)
In the past month, did you have…					
…any pain in your mouth?	0 (0.0)	0 (0.0)	0 (0.0)	0 (0.0)	2 (100.0)
…a feeling of uncertainty about your teeth, mouth, or dentures?	0 (0.0)	0 (0.0)	1 (50.0)	0 (0.0)	1 (50.0)

Brittle Cornea Syndrome

OHIP-14	Never	Hardly ever	Occasio nally	Often	Very often
Have you had any problems with your teeth, mouth or dentures in the past month…					
…making it difficult to pronounce certain words?	0 (0.0)	0 (0.0)	0 (0.0)	1 (100.0)	0 (0.0)
…that made you feel that your sense of taste was impaired?	0 (0.0))	0 (0.0)	0 (0.0)	1 (100.0)	0 (0.0)
…that gave you the impression that your life in general was less satisfying?	0 (0.0)	0 (0.0)	0 (0.0)	1 (100.0)	0 (0.0)
…making it difficult to relax?	0 (0.0)	0 (0.0)	0 (0.0)	0 (0.0)	1 (100.0)
Has it often happened in the past month due to problems with your teeth, mouth or dentures…					
…that you felt tense?	0 (0.0)	0 (0.0)	0 (0.0)	0 (0.0)	1 (100.0)
…that you had to interrupt meals?	0 (0.0)	0 (0.0)	0 (0.0)	0 (0.0)	1 (100.0)
…that you were uncomfortable eating certain foods?	0 (0.0)	0 (0.0)	0 (0.0)	0 (0.0)	1 (100.0)
…that you have been a bit irritable with other people?	0 (0.0)	0 (0.0)	0 (0.0)	0 (0.0)	1 (100.0)
…that you had difficulty doing your usual jobs?	0 (0.0)	0 (0.0)	0 (0.0)	0 (0.0)	1 (100.0)
…that you were completely unable to do anything?	0 (0.0)	0 (0.0)	0 (0.0)	1 (100.0)	0 (0.0)
…that you have been a bit embarrassed?	0 (0.0)	0 (0.0)	0 (0.0)	0 (0.0)	1 (100.0)
…that your diet has been unsatisfactory?	0 (0.0)	0 (0.0)	1 (100.0)	0 (0.0)	0 (0.0)
In the past month, did you have…					
…any pain in your mouth?	0 (0.0)	0 (0.0)	0 (0.0)	0 (0.0)	1 (100.0)
…a feeling of uncertainty about your teeth, mouth, or dentures?	0 (0.0)	0 (0.0)	0 (0.0)	0 (0.0)	1 (100.0)

Myopathic EDS

OHIP-14	Never	Hardly ever	Occasio nally	Often	Very often
Have you had any problems with your teeth, mouth or dentures in the past month…					
…making it difficult to pronounce certain words?	2 (100.0)	0 (0.0)	0 (0.0)	0 (0.0)	0 (0.0)
…that made you feel that your sense of taste was impaired?	1 (50.0)	0 (0.0)	0 (0.0)	1 (50.0)	0 (0.0)
…that gave you the impression that your life in general was less satisfying?	0 (0.0)	0 (0.0)	0 (0.0)	1 (50.0)	1 (50.0)
…making it difficult to relax?	0 (0.0)	0 (0.0)	0 (0.0)	1 (50.0)	1 (50.0)
Has it often happened in the past month due to problems with your teeth, mouth or dentures…					
…that you felt tense?	0 (0.0)	0 (0.0)	0 (0.0)	0 (0.0)	2 (100.0)
…that you had to interrupt meals?	0 (0.0)	0 (0.0)	0 (0.0)	1 (50.0)	1 (50.0)
…that you were uncomfortable eating certain foods?	0 (0.0)	0 (0.0)	0 (0.0)	1 (50.0)	1 (50.0)
…that you have been a bit irritable with other people?	0 (0.0)	0 (0.0)	0 (0.0)	2 (100.0)	0 (0.0)
…that you had difficulty doing your usual jobs?	0 (0.0)	0 (0.0)	0 (0.0)	2 (100.0)	0 (0.0)
…that you were completely unable to do anything?	1 (50.0)	0 (0.0)	0 (0.0)	1 (50.0)	0 (0.0)
…that you have been a bit embarrassed?	1 (50.0)	1 (50.0)	0 (0.0)	0 (0.0)	0 (0.0)
…that your diet has been unsatisfactory?	0 (0.0)	0 (0.0)	0 (0.0)	0 (0.0)	2 (100.0)
In the past month, did you have…					
…any pain in your mouth?	0 (0.0)	0 (0.0)	0 (0.0)	0 (0.0)	2 (100.0)
…a feeling of uncertainty about your teeth, mouth, or dentures?	0 (0.0)	0 (0.0)	0 (0.0)	0 (0.0)	2 (100.0)

Periodontal EDS

OHIP-14	Never	Hardly ever	Occasio nally	Often	Very often
Have you had any problems with your teeth, mouth or dentures in the past month…					
…making it difficult to pronounce certain words?	3 (100.0)	0 (0.0)	0 (0.0)	0 (0.0)	0 (0.0)
…that made you feel that your sense of taste was impaired?	2 (66.7)	0 (0.0)	1 (33.3)	0 (0.0)	0 (0.0)
…that gave you the impression that your life in general was less satisfying?	1 (33.3)	0 (0.0)	1 (33.3)	1 (33.3)	0 (0.0)
…making it difficult to relax?	1 (33.3)	0 (0.0)	0 (0.0)	2 (66.7)	0 (0.0)
Has it often happened in the past month due to problems with your teeth, mouth or dentures…					
…that you felt tense?	1 (33.3)	0 (0.0)	0 (0.0)	2 (66.7)	0 (0.0)
…that you had to interrupt meals?	1 (33.3)	1 (33.3)	0 (0.0)	1 (33.3)	0 (0.0)
…that you were uncomfortable eating certain foods?	1 (33.3)	0 (0.0)	1 (33.3)	1 (33.3)	0 (0.0)
…that you have been a bit irritable with other people?	1 (33.3)	0 (0.0)	1 (33.3)	1 (33.3)	0 (0.0)
…that you had difficulty doing your usual jobs?	1 (33.3)	0 (0.0)	1 (33.3)	1 (33.3)	0 (0.0)
…that you were completely unable to do anything?	2 (66.7)	0 (0.0)	0 (0.0)	1 (33.3)	0 (0.0)
…that you have been a bit embarrassed?	1 (33.3)	1 (33.3)	0 (0.0)	1 (33.3)	0 (0.0)
…that your diet has been unsatisfactory?	2 (66.7)	0 (0.0)	1 (33.3)	0 (0.0)	0 (0.0)
In the past month, did you have…					
…any pain in your mouth?	0 (0.0)	0 (0.0)	1 (33.3)	1 (33.3)	1 (33.3)
…a feeling of uncertainty about your teeth, mouth, or dentures?	0 (0.0)	0 (0.0)	1 (33.3)	1 (33.3)	1 (33.3)
