# Supplementary material for: Oral Health-Related Quality of Life in Different Subtypes of Ehlers-Danlos Syndrome
Source: Int J Environ Res Public Health. 2023 Jan 26;20(3):2218. doi: 10.3390/ijerph20032218 (PMC9916345; doi:10.3390/ijerph20032218)
Supplement: Supplementary file 1 [file ijerph-20-02218-s001.zip › ijerph-2122009-supplementary/Supplementary Table S4.pdf]

Supplementary Table S4

Effect sizes (Cohen´s d; 95% confidence interval) for the comparison of Oral Health Impact Profile (OHIP-14) Scores are shown in the lower half of each table. Significant values are bold; Cohen´s d and confidence interval not given for the comparison of cardiac-vascular EDS (cvEDS) and Brittle Cornea Syndrome (BCS), only one participant respectively; n.a. = not applicable

ORAL FUNCTION

	cEDS	clEDS	cvEDS	vEDS	hEDS	aEDS	kEDS	BCS	mEDS	pEDS
cEDS	-									
clEDS	-3.7 (-1.20 – 0.46)	-								
cvEDS	0.59 (-1.42 – 2.58)	1.16 (-1.08 – 3.31)	-							
vEDS	0.35 (-0.25 – 0.94)	0.79 (-0.12 – 1.68)	-0.28 (-2.29 – 1.74)	-						
hEDS	-0.08 (-0.46 – 0.31)	0.31 (-0.44 – 1.06)	-0.70 (-2.67 – 1.27)	-0.43 (-0.91 – 0.05)	-					
aEDS	0.60 (-0.85 – 2.03)	1.20 (-0.53 – 2.85)	0.00 (-2.40 – 2.40)	0.29 (-1.18 – 1.75)	0.70 (-0.69 – 2.10)	-				
kEDS	-0.85 (-2.30 – 0.60)	-0.54 (-2.11 – 1.08)	-0.99 (-3.52 – 1.83)	-1.27 (-2.77 – 0.26)	-0.84 (-2.24 – 0.55)	-1.30 (-3.49 – 1.09)	-			
BCS	-1.50 (-3.51 – 0.55)	-1.37 (-3.55 – 0.90)	n.a.	-2.02 (-4.12 – 0.13)	-1.51 (-3.48 – 0.46)	-3.54 (-8.39 – 1.16)	-0.42 (-2.80 – 2.11)	-		
mEDS	**-2.16 (-3.67 - -0.60)**	**-2.28 (-4.19 - -0.27)**	-9.19 (-20.79 – 0.47)	**-2.78 (-4.47 - -1.04)**	**-2.17 (-3.58 - -0.77)**	**-5.81 (-11.45 - -0.39)**	-1.18 (-3.31 – 1.15)	-2.12 (-5.49 – 1.46)	-	
pEDS	0.17 (-1.02 – 1.36)	0.59 (-0.81 – 1.96)	-0.38 (-2.63 – 1.95)	-0.18 (-1.40 – 1.05)	0.26 (-0.88 – 1.40)	-0.43 (-2.22 – 1.42)	0.84 (-1.12 – 2.68)	1.51 (-1.25 – 4.04)	2.50 (-0.18 – 5.04)	-

OROFACIAL PAIN

	cEDS	clEDS	cvEDS	vEDS	hEDS	aEDS	kEDS	BCS	mEDS	pEDS
cEDS	-									
clEDS	**-0.86 (-1.71- -0.01)**	-								
cvEDS	0.66 (-1.35 – 2.66)	1.83 (-0.56 – 4.09)	-							
vEDS	0.25 (-0.34 – 0.84)	**1.25 (0.29 – 2.18)**	-048 (-2.49 – 1.55)	-						
hEDS	-0.21 (-0.60 – 0.17)	**0.85 (0.10 – 1.61)**	-1.07 (-3.04 – 0.90)	**-0.52 (-1.01 - -0.4)**	-					
aEDS	0.48 (-0.96 – 1.92)	1.62 (-0.20 – 3.36)	-0.24 (-2.61 – 2.23)	0.28 (-1.19 – 1.74)	0.84 (-0.56 – 2.23)	-				
kEDS	-0.97 (-2.42 – 0.49)	-0.16 (-1.73 – 1.42)	-2.12 (-5.49 – 1.46)	-1.38 (-2.89 – 0.17)	-1.02 (-2.42 – 0.38)	-1.89 (-4.39 – 0.81)	-			
BCS	-1.51 (-3.53 – 0.54)	-0.82 (-2.93 – 1.36)	n.a.	-1.99 (-4.08 – 0.16)	-1.72 (-3.69 – 0.25)	-2.59 (-6.43 – 1.35)	-0.71 (-3.13 -1.96)	-		
mEDS	-1.35 (-2.81 – 0.14)	-0.64 (-2.23 – 0.99)	-7.78 (-17.65 – 0.60)	**-1.83 (-3.38 - -0.23)**	**-1.49 (-2.89 - -0.09)**	-3.16 (-6.57 – 0.34)	-0.63 (-2.61 -1.48)	0.71 (-1.96 – 3.13)	-	
pEDS	-0.31 (-1.50 – 0.88)	0.70 (-0.71 – 2.07)	-1.75 (-4.39 – 1.14)	-0.64 (-1.87 – 0.61)	-0.17 (-1.31 – 0.97)	-1.24 (-3.19 – 0.86)	1.05 (-0.98 – 2.94)	2.18 (-0.94 – 5.08)	2.16 (-0.35 – 4.51)	-

OROFACIAL APPEARANCE

	cEDS	clEDS	cvEDS	vEDS	hEDS	aEDS	kEDS	BCS	mEDS	pEDS
cEDS	-									
clEDS	-0.63 (-1.46 – 0.22)	-								
cvEDS	-1.21 (-3.22 – 0.82)	-0.45 (-2.54 – 1.68)	-							
vEDS	0.32 (-0.27 – 0.91)	0.87 (-0.05 – 1.77)	1.40 (-0.68 – 3.45)	-						
hEDS	-0.80 (-0.47 – 0.31)	0.50 (-0.26 – 1.25)	1.00 (-0.97 – 2.96)	-0.38 (-0.86 – 0.11)	-					
aEDS	0.48 (-0.96 – 1.92)	0.87 (-0.79 – 2.48)	0.99 (-1.83 – 3.52)	0.14 (-1.32 – 1.60)	0.52 (-0.88 – 1.91)	-				
kEDS	-0.49 (-1.92 – 0.96)	0.15 (-1.43 – 1.72)	2.12 (-1.46 – 5.49)	-0.76 (-2.23 – 0.73)	-0.35 (-1.74 – 1. 05)	-0.78 (-2.79 – 1.38)	-			
BCS	**-2.19 (-4.25 - -0.10)**	-1.24 8-3.39 – 1.01)	n.a.	**-2.29 (-4.42 - -0.11)**	-1.86 (-3.83 – 0.11)	-1.56 (-4.44 – 1.62)	-4.95 (-11.43 – 0.94)	-		
mEDS	**-2.23 (-3.76 - -0.67)**	-1.33 (-3.01 – 0.42)	n.a.	**-2.36 (-3.98 - -0.68)**	**-1.86 (-3.26 - -0.46)**	-2.20 (-4.90 – 0.69)	**-7.00 (-13.68 - -0.66)**	n.a.	-	
pEDS	-0.71 (-1.91 – 0.50)	-0.06 (-1.41 – 1.30)	0.38 (-1.95 – 2.63)	-0.94 (-2.19 – 0.33)	-0.56 (-1.70 – 0.58)	-0.84 (-2.68 – 1.12)	-0.23 (-2.01 – 1.59)	1.13 (-1.46 – 3.52)	1.39 (-0.77 – 3.39)	-

PSYCHOSOCIAL IMPACT

	cEDS	clEDS	cvEDS	vEDS	hEDS	aEDS	kEDS	BCS	mEDS	pEDS
cEDS	-									
clEDS	-0.46 (-1.29 – 0.38)	-								
cvEDS	-0.55 (-2.54 – 1.46)	-0.06 (-2.15 – 2.04)	-							
vEDS	-0.07 (-0.66 – 0.52)	0.41 (-0.47 – 1.29))	0.52 (-1.51 – 2.54)	-						
hEDS	-0.02 (-0.37 – 0.41)	0.56 (-0.19 – 1.31)	0.65 (-1.32 – 2.61)	0.10 (-0.38 – 0.58)	-					
aEDS	-0.30 (-1.73 – 1.14)	0.13 (-1.44 – 1.70)	0.14 (-2.29 – 2.52)	-0.25 (-1.71 – 1.22)	-0.37 (-1.76 – 1.02)	-				
kEDS	-0.56 (-1.99 – 0.89)	-0.06 (-1.63 – 1.51)	n.a.	-0.54 (-2.00 – 0.94)	-0.65 (-2.04 – 0.75)	-0.20 (-2.15 – 1.79)	-			
BCS	-1.99 (-4.03 – 0.09)	-1.27 (-3.43 – 0.99)	n.a.	-2.10 (-4.20 – 0.06)	**-2.29 (-4.26 - -0.31)**	-0.99 (-3.52 – 1.83)	n.a.	-		
mEDS	-1.49 (-2.96 – 0.01)	-0.85 (-2.45 – 0.81)	-0.71 (-3.13 – 1.96)	-1.52 (-3.04 – 0.04)	**-1.74 (-3.13 - -0.33)**	-0.78 (-2.79 – 1.38)	-1.00 (-3.07 – 1.25)	0.35 (-2.15 – 2.72)	-	
pEDS	-0.38 (-1.57 – 0.82)	0.08 (-1.28 – 1.43)	0.13 (-2.15 – 2.38)	-0.34 (-1.56 – 0.90)	-0.46 (-1.60 – 0.68)	-0.06 (-1.84 – 1.74)	0.16 (-1.64 – 1.94)	1.33 (-1.35 – 3.78)	0.89 (-1.08 – 2.74)	-
